# Per-patient Comparison of [^68^Ga]-FAPI-46 Versus [^18^F]FDG as PET Tracers in ER-positive Breast Cancer: Study Design of a Single-centre Prospective Study

**DOI:** 10.7150/jca.138448

**Published:** 2026-09-18

**Authors:** Melissa Lenaerts, Lisa E.H.W. Duijx, Felix M. Mottaghy, Matthias Bauwens, Robin M.J.M. van Geel, Vivianne C.G. Tjan-Heijnen, Philipp Backhaus, Loes F.S. Kooreman, Patty J. Nelemans, Marjolein L. Smidt, Joachim E. Wildberger, Thiemo J.A. van Nijnatten

**Affiliations:** 1 Department of Surgery, Maastricht University Medical Centre+, Maastricht, The Netherlands.; 2 GROW - Research Institute for Oncology and Reproduction, Maastricht University, Maastricht, The Netherlands.; 3 Department of Radiology and Nuclear Medicine, Maastricht University Medical Centre+, Maastricht, The Netherlands.; 4 Department of Nuclear Medicine, University Hospital RWTH Aachen University, Aachen, Germany.; 5 Centre of Integrated Oncology Aachen Bonn Cologne Duesseldorf (CIO ABCD), Aachen, Germany.; 6 NUTRIM Research Institute, Maastricht University, Maastricht, The Netherlands.; 7 Department of Clinical Pharmacy and Toxicology, Maastricht University Medical Centre+, Maastricht, The Netherlands.; 8 Department of Medical Oncology, Maastricht University Medical Centre+, Maastricht, The Netherlands.; 9 Department of Nuclear Medicine, University Hospital Munster, Münster, Germany.; 10 European Institute for Molecular Imaging, University of Munster, Münster, Germany.; 11 Department of Pathology, Maastricht University Medical Centre+, Maastricht, The Netherlands.; 12 Department of Epidemiology, Maastricht University, Maastricht, The Netherlands.; 13 CARIM - Cardiovascular Research Institute Maastricht, Maastricht University, Maastricht, The Netherlands.; 14 Dutch Expert Centre for Screening (LRCB), Nijmegen, The Netherlands.

**Keywords:** breast neoplasms, receptors, estrogen, FAPI-46, positron emission tomography computed tomography, discordant lesions

## Abstract

**Introduction.** [^18^F]FDG can be considered as PET tracer to determine whole-body staging in patients with locally advanced (LABC), metastatic, and recurrent breast cancer. Yet, previous studies demonstrated a lower amount of metabolic accumulation on [^18^F]FDG PET in case of oestrogen receptor (ER)-positive breast cancer. Consequently, given the lower metabolic accumulation, patients with ER-positive breast cancer might be at risk for underdiagnosis according to [^18^F]FDG PET. A novel PET tracer, [^68^Ga]-fibroblast activation protein inhibitor (FAPI)-46, has shown promising results in breast cancer (re)staging. This single-centre prospective pilot study aims to explore the relative diagnostic accuracy of [^68^Ga]-FAPI-46 compared to [^18^F]FDG in patients with ER-positive breast cancer and will provide the required information to support sample size calculation for a future validation study.

**Methods.** In this study, 20 patients with ER-positive LABC, metastatic, or recurrent breast cancer will undergo the standard [^18^F]FDG PET exam and a [^68^Ga]-FAPI-46 PET/CT and [^68^Ga]-FAPI-46 PET/MRI exam. Histopathological confirmation will be obtained in cases of discordant or concordant (double-positive) findings, depending on clinical relevance and technical feasibility. The study will provide preliminary estimates of the relative accuracy of [^68^Ga]-FAPI-46 PET when compared to [^18^F]FDG PET as well as the proportion of discordant findings and patients in whom a biopsy is feasible.

**Conclusions.** The present single-centre prospective pilot study will characterize the potential of [^68^Ga]-FAPI-46 PET to identify additional lesions not detected on a [^18^F]FDG PET exam in patients with ER-positive breast cancer to estimate an adequate sample size for a future validation study.

**Trial registration.** This study was registered prospectively on ClinicalTrials.gov. Trial registration number: NCT06335069; Register date: 28 March 2024.

## Introduction

Breast cancer is the most common type of invasive cancer among women, with more than 2.4 million new diagnoses worldwide in 2024 1. The prognosis of breast cancer patients is strongly influenced by the extent of the disease 2. The five-year survival rate is 100% when confined to the breast, 87.2% with axillary lymph node involvement, and 32.6% with distant metastases 3. Consequently, patients with more extensive disease at diagnosis, such as those with locally advanced breast cancer (LABC), require more comprehensive staging at diagnosis. LABC is typically defined as a primary tumour larger than 5 cm and/or the presence of lymph node metastases (cT3-4N0 or cT1-4N+) 4. Patients with LABC, metastatic breast cancer, or recurrent breast cancer receive distant staging using [^18^F]-fluorodeoxyglucose ([^18^F]FDG) positron emission tomography (PET) 5. [^18^F]FDG PET is combined with either computed tomography (CT) or magnetic resonance imaging (MRI) to obtain morphologic information 2.

Previous studies have shown that the amount of metabolism on [^18^F]FDG PET varies between breast cancer clinical subtypes, with a significantly lower metabolic activity in the oestrogen receptor (ER)-positive subtype 6-8. Furthermore, Iqbal *et al*. assessed the diagnostic performance of [^18^F]FDG PET in a cohort of 70 ER-positive grade 1-2 breast cancer patients prior to treatment. Of the 155 lesions analysed, 7 (4.5%) were false negatives, affecting 7 patients, while 17 lesions (11%) were false positives in 9 patients. [^18^F]FDG PET staging of grade 1-2 ER-positive breast cancer resulted in misclassification in 22.9% of patients 8. The lower metabolic activity in ER-positive, low-grade tumours can have important diagnostic consequences, as low FDG-avidity may lead to missed metastatic locations, potentially resulting in incorrect staging and treatment decisions 9-11.

Given these limitations of [^18^F]FDG in ER-positive breast cancer, there is a need for an alternative PET tracer to improve baseline staging. A promising option is the fibroblast activation protein inhibitor (FAPI), which binds to fibroblast activation protein, a type II transmembrane serine protease expressed on the surface of activated cancer-associated fibroblasts within the tumour stroma (Figure 1). Fibroblast activation protein is highly expressed in more than 90% of epithelial-derived tumours and their metastases, whereas not expressed in healthy tissue, benign lesions, and precancerous tissues 12. This characteristic makes it a potential target for both diagnosing and treating malignant tumours 12. FAPI can be radiolabelled with Gallium-68 ([^68^Ga]) or with Fluorine-18 ([^18^F]), creating the ^68^Ga-FAPI or ^18^F-FAPI PET tracers. Previous studies with ^68^Ga-FAPI and ^18^F-FAPI have demonstrated enhanced lesion detectability, higher standardised uptake values (SUV) and improved tumour-to-background-ratios (TBR) across multiple cancer types, including breast cancer 13-17. However, a direct comparison of ^68^Ga-FAPI PET and [^18^F]FDG PET in ER-positive breast cancer patients specifically has not yet been conducted.

Therefore, to address this gap, we propose a study design for a single-centre prospective pilot study to compare the relative diagnostic accuracy of [^68^Ga]-FAPI-46 and [^18^F]FDG as PET tracers in patients with ER-positive breast cancer.

## Methods

### Study design and patient selection

This single-centre prospective pilot study will compare the relative diagnostic accuracy of [^68^Ga]-FAPI-46-PET and [^18^F]FDG PET in patients with ER-positive breast cancer. All included patients will undergo [^68^Ga]-FAPI-46 PET/CT and [^68^Ga]-FAPI-46 PET/MRI in addition to their standard [^18^F]FDG PET exam. Histological results from biopsy will be used as reference standard. Biopsy will only be performed in patients who test positive on at least one tracer, verification of disease status in patients who test negative is not possible in the time frame of our study. This study will include female patients diagnosed with histopathological proven ER-positive LABC (cT3-4N0 or cT1-4N+), metastatic breast cancer, or recurrent breast cancer regardless of HER2 status who are scheduled to receive a whole-body [^18^F]FDG PET scan for distant staging (Table 1). Based on feasibility considerations, a total of 20 patients will be included, recruited at the Maastricht UMC+ (Maastricht, the Netherlands). Informed consent will be written, dated, and signed by the investigator and all participants. The study has been approved by the Medical Research Ethics Committee of Maastricht UMC+ via the clinical trial information system (CTIS; 2023-508066-15) and is registered at ClinicalTrials.gov (Identifier: NCT06335069). Monitoring will be conducted by the independent party Clinical Trial Centre Maastricht (CTCM).

### Radiopharmaceutical protocol

The precursor, FAPI-46 (SOFIE, Dulles, VA, USA), is labelled with the radioactive isotope [^68^Ga] at the radiopharmacy facility in Maastricht UMC+, following GMP-z compliant protocols and using an automated synthesis module (Scintomics, Germany). In short, [^68^Ga] is eluted from a [^68^Ge]/[^68^Ga] generator (Eckert-Ziegler) using 0.1 M HCl, adjusted to pH 3.5 using a HEPES buffer and combined with 50 microgram FAPI-46 precursor at 95 °C for 10 minutes. After synthesis, [^68^Ga]-FAPI-46 is purified using a Sep-PakC18 mini cartridge, where it is collected using 2 ml Ethanol/water (50/50) and diluted with 12 ml sterile PBS solution containing 0.1 mg ascorbic acid. Prior to injection, quality control of each radiosynthesis is performed, consisting of HPLC (identity, radiochemical and chemical purity), TLC (radiochemical purity), endotoxin test, pH and visual appearance.

### Imaging protocol

All included patients will undergo [^68^Ga]-FAPI-46 PET/CT and [^68^Ga]-FAPI-46 PET/MRI in addition to their standard [^18^F]FDG PET exam. The time between the [^18^F]FDG PET exam and [^68^Ga]-FAPI-46 PET exam is required to be at least 48 hours to prevent potential interference from residual tracer activity. The maximum permitted time interval between scans will be 20 working days. To perform the [^68^Ga]-FAPI-46 PET/CT and PET/MRI scan, patients receive an intravenous injection of [^68^Ga]-FAPI-46 at a dose of 3 MBq/kg with a minimum of 100 MBq. The [^68^Ga]-FAPI-46 PET/CT scan will take place after a waiting period of 30 minutes, immediately followed by the [^68^Ga]-FAPI-46 PET/MRI scan 15, 18. Neither exam involved the administration of a contrast agent. After acquisition, the [^68^Ga]-FAPI-46 PET scans will be evaluated for discordant lesions compared to the [^18^F]FDG PET scan. Based on this initial clinical assessment, a biopsy will be performed if clinically indicated and technically feasible, after discussion in the multidisciplinary team meeting. After completion of the study, two independent readers will re-evaluate all [^18^F]FDG PET and [^68^Ga]-FAPI-46 PET exams in a randomised order, assessing for parameters such as quantitative metabolic measurements and number of suspected lesions. This will be performed with a 8-week interval between [^68^Ga]-FAPI-46 and [^18^F]FDG assessments to minimize recall bias. Afterwards, both readers come together to reach consensus on their evaluations.

### Histopathological biopsy

Histopathological biopsy will be considered for additional suspicious lesions detected during the PET exams, but not for the already histopathologically confirmed primary breast tumour. The decision for biopsy will be made on a lesion-by-lesion basis, depending on the tracers uptake pattern and the potential impact on clinical management. Specifically, biopsy will be considered in cases where the final interpretation indicates discordance between [^68^Ga]-FAPI-46 and [^18^F]FDG ([^18^F]FDG-positive and [^68^Ga]-FAPI-46-negative or [^18^F]FDG-negative and [^68^Ga]-FAPI-46-positive) or when an additional lesion is positive on both tracers but has not been previously confirmed. Whether a biopsy will be performed, will depend on two relevant conditions:

1. Does the additional lesion change the treatment choice? If the patient is already diagnosed with six or more hypermetabolic distant metastases according to [^18^F]FDG PET, no biopsy will be performed.

2. Technical feasibility of performing a biopsy. In some rare cases, depending on the anatomical location of the hypermetabolic lesion, it may be impossible to perform a biopsy. In such instances, these lesions will be mentioned for follow-up, with the duration of follow-up determined in consultation with clinicians.

Histopathological biopsy will be handled according to the standard of care.

### Statistical analysis

This pilot study (n=20) will inform sample size estimation for a future validation study. As biopsy can only be performed in patients testing positive on at least one tracer, complete disease verification is not feasible. Longer follow-up will therefore be needed in subsequent studies. Nonetheless, 20 patients are sufficient to provide meaningful preliminary estimates 19. Specifically, we will estimate**:** (1) the proportion of discordant pairs; (2) the proportion of lesions where biopsy is indicated and feasible; (3) the proportion of [^68^Ga]-FAPI-46-positive and/or [^18^F]FDG-positive lesions confirmed to have a histologically positive or negative lesion (gold standard); and (4) the proportion of lesions detected on [^68^Ga]-FAPI-46 PET/CT vs. [^68^Ga]-FAPI-46 PET/MRI, which will be calculated using the number of lesions detected by one specific modality divided by the total number of lesions detected by both modalities. The proportions (3) will be used for preliminary estimates of the relative true positive fraction and false positive fraction when comparing [^68^Ga]-FAPI-46 and [^18^F]FDG PET.

The analyses will be performed on a patient level. Statistical analysis will be performed using the Statistical Package for the Social Sciences (V28, IBM Corp, Armonk, New York, USA).

## Discussion

Accurate staging of LABC (cT3-4N0 or cT1-4N+), metastatic-, and recurrent breast cancer, particularly in the ER-positive subtype, remains challenging due to the limitations of the current staging method by using [^18^F]FDG as PET tracer. ER-positive breast cancer lesions often exhibit lower metabolic activity on [^18^F]FDG PET compared to other breast cancer clinical subtypes, making this subgroup less suitable for staging using [^18^F]FDG PET as it may result in underestimation of the disease extent 7, 8, 20. Given this limitation, alternative PET-tracers such as ^68^Ga-FAPI may provide a more effective solution, particularly for patients with ER-positive breast cancer. Consequently, we propose a single-centre prospective pilot study to compare the relative diagnostic accuracy of [^68^Ga]-FAPI-46 and [^18^F]FDG as PET tracers in patients diagnosed with ER-positive breast cancer.

FAPI is a relatively novel PET tracer in oncologic imaging. Therefore, in recent years, several studies have compared its diagnostic efficacy with [^18^F]FDG, the current standard of care 21, 22. Multiple review articles across various cancer types revealed FAPI PET to offer superior sensitivity and specificity compared to [^18^F]FDG PET, with greater lesion detectability 23-26. Moreover, [^18^F]FDG shows nonspecific uptake in crucial organs, whereas ^68^Ga-FAPI shows a drastic reduction of the background signal in the brain, liver, oro-and nasopharyngeal mucosa, and the gastrointestinal tract, resulting in a higher TBR 27. In addition, previous studies concerning ^68^Ga-FAPI in breast cancer, not solely focusing on the ER-positive breast cancer subtype, have reported a higher detection rate and higher SUV values for ^68^Ga-FAPI PET compared to [^18^F]FDG PET within the breast, axillary lymph nodes, and distant metastases 15-17.

Despite the promising preliminary evidence, no prospective study has specifically compared FAPI PET with [^18^F]FDG PET in ER-positive breast cancer. To address this gap, this study is designed to provide insights on this topic. The primary aim of this pilot study is to obtain data on the relative performance of both tracers and the feasibility of taking biopsies in test-positive patients. If FAPI PET detects more lesions compared to [^18^F]FDG PET, it may be considered superior, however, it would not be preferable if this comes at the cost of a higher rate of false-positive results. The results of this study will be valuable for assessing the feasibility of a larger, potentially multicentre study, and identifying potential challenges in the study workflow. While this pilot study is not intended to detect subtle statistical differences, it will generate insights into essential input parameters for an adequate sample size calculation for a diagnostic study that compares the diagnostic accuracy of [^18^F]FDG PET and [^68^Ga]-FAPI-46 as PET tracers in patients with ER-positive breast cancer.

A major strength of the study design is its prospective approach, which allows for systematic data collection and minimises the risk of bias. The use of standardised imaging protocols increases reproducibility and ensures consistent acquisition across patients. Furthermore, the blinded image interpretation limits observer-related variability and strengthens the reliability of the results. Finally, the single-centre setting will facilitate strict protocol adherence, supporting strong internal validation.

Despite these strengths, several limitations should be acknowledged. An important limitation of ^68^Ga-FAPI is the potential of false positive findings, since non-malignant fibrotic processes and chronic inflammatory diseases such as fibroadenoma, liver fibrosis, and rheumatoid arthritis are associated with fibroblast activity, and may exhibit ^68^Ga-FAPI uptake 17, 28. However, this potential limitation is partly mitigated by the integration of multimodal imaging in this study ([^18^F]FDG PET, CT, and MRI), which provides an opportunity to further characterize such lesions. By correlating these findings with histopathology when available, the imaging data may help to distinguish benign from malignant uptake patterns. This approach could reduce the need for unnecessary biopsies, as lesions suspected to represent false positives may be identified through imaging characteristics alone. Although multimodal imaging is an appropriate strategy for the current study, the independent use of ^68^Ga-FAPI PET as a stand-alone imaging modality could be more limited, given the reduced ability to correlate uptake with anatomical or other functional imaging findings.

Nonetheless, a key remaining limitation of this study is that when a lesion appears negative on the ^68^Ga-FAPI scan, no biopsy will be performed. As a result, false-negative and true-negative findings cannot be estimated, and sensitivity, specificity, NPV, and accuracy remain unknown. Only the relative accuracy of ^68^Ga-FAPI and [^18^F]FDG can be estimated 19. Future research may provide further insights by incorporating longer follow-up.

In addition to comparing the relative diagnostic accuracy of [^68^Ga]-FAPI-46 with [^18^F]FDG, this study will provide essential feasibility data, optimise scanning protocols, and identify potential logistical or methodological obstacles, laying the groundwork for a diagnostic study. In particular, by performing ^68^Ga-FAPI PET/CT immediately followed by ^68^Ga-FAPI PET/MRI with a single tracer injection, the study will generate insights not only into the imaging parameters, but also into the practical workflow, timing, and coordination required to acquire high-quality images across two modalities. Eventually, these results will guide future imaging strategies in ER-positive breast cancer, potentially impacting lesion detection, staging accuracy, and treatment planning. Based on the results of this pilot study, including the relative accuracy and available histopathology, it will be possible to estimate the number of patients required for a future validation study to robustly assess sensitivity, specificity, and the feasibility of taking biopsies.

## Conclusion

[^68^Ga]-FAPI-46 is a highly promising novel PET tracer, though it has not yet been investigated specifically in patients with ER-positive breast cancer. This single-centre prospective pilot study will compare the relative diagnostic accuracy of [^68^Ga]-FAPI-46 and [^18^F]FDG as PET tracer in patients with ER-positive LABC, metastatic, and recurrent breast cancer.

## Figures and Tables

**Figure 1:**
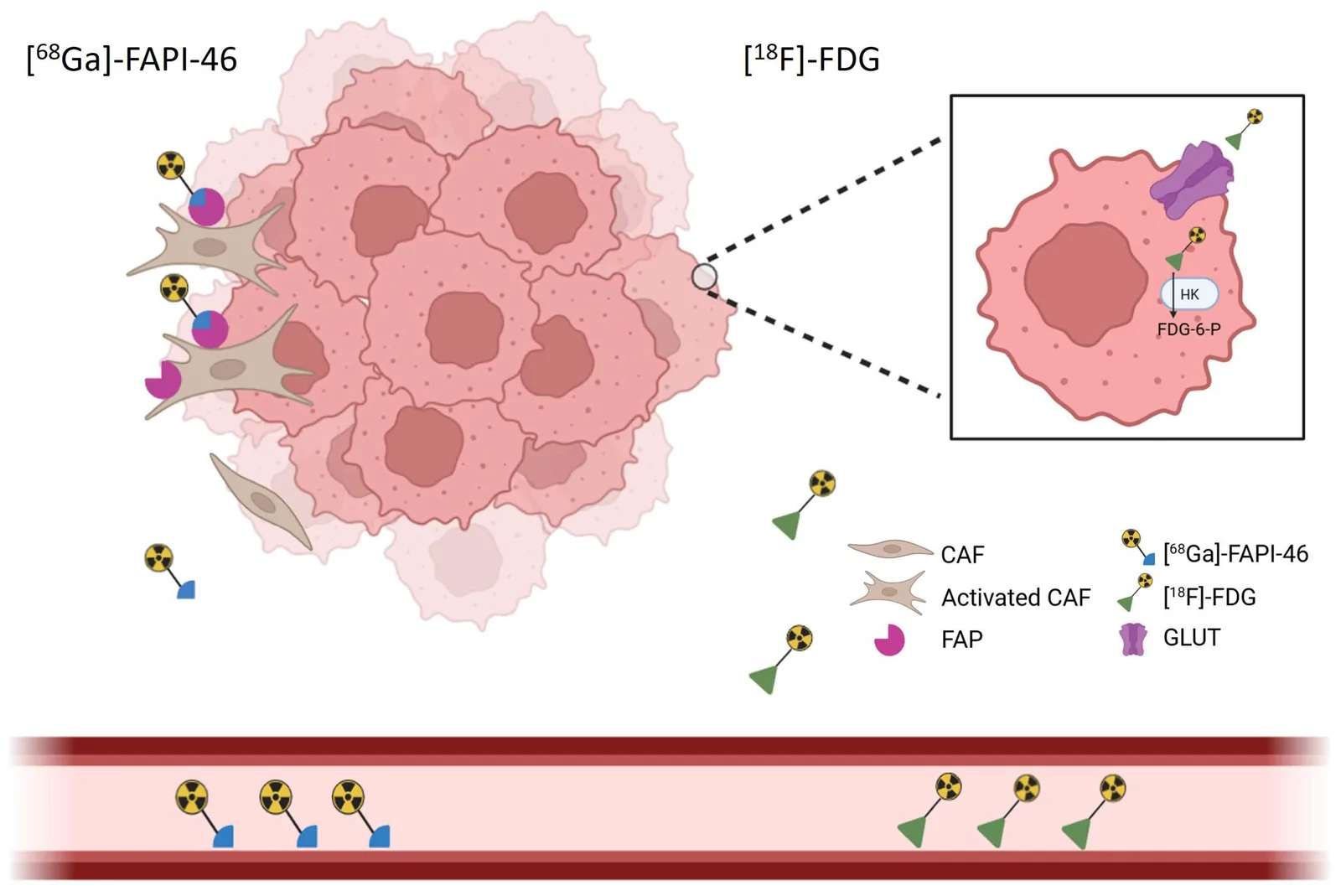
Schematic illustration of the working principles of [^68^Ga]-FAPI-46 (left) and [^18^F]FDG (right) as PET tracers. [^68^Ga]-FAPI-46 binds to fibroblast activation protein (FAP), expressed by cancer-associated fibroblasts in the tumour microenvironment. Created with www.biorender.com.

**Table 1:** Eligibility criteria

**Inclusion criteria**
Female patient with histopathological proven ER-positive breast cancer (LABC (cT3-4N0 or cT1-4N+), metastatic, or recurrent breast cancer), regardless of HER2 status, at diagnosis
Distant staging by [^18^F]FDG PET/CT or [^18^F]FDG PET/MRI
**Exclusion criteria**
Age <18
Pregnancy
Secondary malignancies
No [^18^F]FDG PET/CT or [^18^F]FDG PET/MRI exam
Contra-indication for PET/MRI (metallic device in body, severe claustrophobia, BMI > 35)
Chronic inflammatory disease (e.g. rheumatoid arthritis)
Severe hepatic or renal impairment (eGFR ≤ 45mL/min/1.73m²) as [^68^Ga]-FAPI-46 is primarily excreted through the kidneys and severe renal impairment may reduce tracer clearance and prolong circulation of the radiopharmaceutical.

## Abbreviations

[^18^F]FDG: [^18^F]-fluorodeoxyglucose
[^68^Ga]: Gallium-68
CT: Computed tomography
CTCM: Clinical trial Centre Maastricht
CTIS: Clinical trial information system
ER: Oestrogen receptor
FAPI: Fibroblast activation protein inhibitor
GMP: Good manufacturing practice
HER2: Human epidermal growth factor receptor 2
LABC: Locally advanced breast cancer
MRI: Magnetic resonance imaging
PET: Positron emission tomography
SUV: Standardised uptake value
TBR: Tumour-to-background ratio
